# Endothelial microparticles: A mechanosensitive regulator of vascular homeostasis and injury under shear stress

**DOI:** 10.3389/fcell.2022.980112

**Published:** 2022-09-12

**Authors:** Shuo Feng, Jia Wei Chen, Xin Yi Shu, Muladili Aihemaiti, Jin Wei Quan, Lin Lu, Rui Yan Zhang, Chen Die Yang, Xiao Qun Wang

**Affiliations:** ^1^ Department of Cardiovascular Medicine, Ruijin Hospital, Shanghai Jiao-Tong University School of Medicine, Shanghai, China; ^2^ Institute of Cardiovascular Disease, Shanghai Jiao-Tong University School of Medicine, Shanghai, China

**Keywords:** shear stress, endothelial microparticles, vascular homeostasis, endothelial dysfunction, atherosclerosis

## Abstract

Hemodynamic shear stress (SS), a frictional force generated by blood flow, regulates vascular homeostasis. High and steady SS maintains physiological function of endothelial cells while low and disturbed SS promotes disturbance of vascular homeostasis and the development of atherosclerosis. Endothelial microparticle (EMP), a vesicular structure shed from endothelial cells, has emerged as a surrogate biomarker of endothelial injury and dysfunction. EMP release is triggered by disturbed SS in addition to multiple inflammatory cytokines. This review systematically summarizes the impact of SS on EMPs and the role of EMPs under SS in modulating vascular homeostasis and injury, including endothelial survival, vasodilation, inflammatory response, vascular permeability, and coagulation system.

## Introduction

Fluid shear stress (SS), a frictional force generated by blood flow, plays a vital role in regulating vascular homeostasis. High and steady SS under laminar flow in the straight part of vessels, is a determinant factor to maintain physiological functions of endothelial cells (ECs) (Heo et al., 2014). By contrast, low and disturbed SS under oscillatory flow at arterial branch points, bifurcations and curvatures, promotes endothelial dysfunction and the pathogenesis of atherosclerosis (Caro et al., 1969).

Endothelial microparticles (EMPs), characterized by phosphatidylserine (PS) externalization, are “medium/large extracellular vesicles” (>200 nm) blebbed from endothelial plasma membrane (Thery et al., 2018). EMPs carry endothelial proteins and some nuclear acids. Given that there is no specific surface maker of EMPs, identification of EMPs generally relies on a combination of maker proteins of different cell types. By using flow cytometry, EMPs are detected and quantified by targeting events with positive EC surface antigens, including CD31 (PECAM-1), CD51 (integrin-αV), CD54 (ICAM-1), CD62e (E-selectin), CD105 (endoglin), CD106 (VCAM-1), CD144 (VE-cadherin), in combination with negative platelet epitopes, such as CD41 and CD42b (Dignat-George and Boulanger, 2011). The specific composition of EMPs is highly dependent on the stimuli and the condition of EC when shedding EMP (Leroyer et al., 2010). EMP release is triggered by a variety of biochemical stimuli, including tumor necrosis factor (TNF) (Jimenez et al., 2003), plasminogen activator inhibitor (PAI) (Brodsky et al., 2002), oxidized low-density lipoprotein (ox-LDL) (Fu et al., 2017), and reactive oxygen species (ROS) (Szotowski et al., 2007). Shedding of EMPs is also stimulated in a mechano-sensitive manner by cyclic stretch (Letsiou et al., 2015), mechanical injury (Andrews et al., 2016), and disturbed SS (Sansone et al., 2018). Mounting evidence has established EMP as a surrogate marker of endothelial dysfunction and atherosclerosis (Lugo-Gavidia et al., 2021).

In this article, we systematically review the impact of SS on EMPs and the role of EMPs in vascular homeostasis and injury under different flow profiles.

## Regulation of EMP release by SS

EMP release is regulated in a mechano-sensitive way that responds differentially to SS of different profiles. In subjects without cardiovascular (CV) disease, plasma EMP levels were inversely associated with brachial artery SS (Vion et al., 2013). A small-scale study showed that low SS provoked by a 30-min distal cuff occlusion intervention resulted in an 83% ± 39% increase in EMPs in healthy adults at sea level (Tremblay et al., 2017). Jenkins et al. also found that forearm occlusion cuff-induced disturbed SS promoted increase of CD62E^+^ EMPs by ∼4 fold and CD31^+^/CD42b^−^ EMPs by ∼9 fold at 20 min, suggesting an acute effect of altered SS on EC apoptosis and EMP release (Jenkins et al., 2013). Besides, EMP release was 2.5-fold higher in cultured ECs exposed to low SS compared to those under high SS (Vion et al., 2013). These studies indicate that low and disturbed SS increases the release of EMPs both *in vivo* and *in vitro*.

The influence of SS on EMP shedding was also observed in patients with established CV disease or risk factors. In patients with end-stage renal disease whose SS was low largely attributed to anemia-related low blood viscosity, EMP levels were inversely associated with SS even after adjustment for age and blood pressure. Of note, elevation of hematocrit levels either by hemodialysis-induced hemoconcentration or erythropoietin anemia improvement led to a significant increase in blood viscosity, rate of SS and finally a significant decrease in EMP levels (Boulanger et al., 2007). Patients with aortic valve stenosis, who presumably had disturbed SS in the ascending aorta, presented a nearly 2-fold higher EMP levels compared to controls (Jung et al., 2017). Hypertensive patients had increased EMP levels as compared to normotensive subjects, and their CD31^+^/41^−^ EMPs were inversely correlated to SS, nitroglycerin-mediated dilation, and diameter of branchial artery (Sansone et al., 2018). Compared to healthy controls, there was also a 3-fold increase of CD62e^+^ EMPs in ischemic stroke patients at acute onset (Chiva-Blanch et al., 2016), who generally had low SS in the carotid artery supplying the affected area (Gnasso et al., 1997; Carallo et al., 2006).

Although the precise mechanism by which SS affects EMP release is still unclear, it appears that the inhibition of EMPs by steady high SS and the induction of EMPs by disturbed low SS are mediated in different pathways. High SS limits EMP release in a nitric oxide (NO)-dependent pathway by inhibiting ABCA1 expression and cytoskeleton reorganization (Vion et al., 2013). High SS also promotes mitochondrial biogenesis through a sirtuin 1 (SIRT1)-dependent mechanism to suppress the release of EMPs (Kim et al., 2015).

On the contrary, disturbed low SS was shown to activate Rho kinases and ERK1/2 pathways, leading to cytoskeletal reorganization and augmented EMP release (Vion et al., 2013). Inflammatory cytokines, such as TNF-α (Combes et al., 1999), lipopolysaccharides, and C-reactive proteins, have been proved to promote EMP release (Dignat-George and Boulanger, 2011). Therefore, disturbed SS may also promote EMP release indirectly by activating EC inflammatory pathways including NF-kB and AP-1 (Helderman et al., 2007).

## Regulation of vascular homeostasis and injury by EMPs under disturbed SS

Findings from *in vivo* and *in vitro* experiments suggest that EMPs are not only markers but also mediators in vascular injury (Jy et al., 2005; Densmore et al., 2006; Abbas et al., 2017; Barak et al., 2017; Mahmoud et al., 2017). Although data exemplifying the specific composition and function of EMPs under SS are still lacking, existing evidence implies that EMPs, either induced by disturbed SS or other stimuli, play multifaceted roles in EC survival, vasodilation, inflammation, barrier function, and coagulation system to disrupt vascular homeostasis at sites of disturbed SS.

### EMPs on EC survival

The impact of EMPs on EC survival is still a matter of debate. EMPs were found to protect ECs from apoptosis by enclosing caspase-3 during vesiculation and diminishing its level in ECs (Abid Hussein et al., 2007). Another study showed that treating ECs with high glucose-derived EMPs significantly increased active caspase-3 in ECs(Bammert et al., 2017). Interestingly, EMPs in a concentrate of 10^5^/ml decreased EC proliferation rate and increased apoptosis rate, whereas “physiological” concentrations of EMPs (10^3^ and 10^4^ EMPs/ml) had no affects (Mezentsev et al., 2005).

Under disturbed SS, ECs exhibit higher turnover rates and enhanced senescence. In human atherosclerotic plaques subjected to disturbed SS, there was pronounced EMP content and senescent makers compared to mammary arteries exposed to steady SS. The collected EMPs promoted premature EC senescence through angiotensin II-induced redox-sensitive activation of mitogen-activated protein kinases and phosphoinositide 3-kinase/Akt, and this phenomenon was only observed when disturbed SS was applied (Abbas et al., 2017).

These data imply a bidirectional impact of EMPs on EC survival. In physiological conditions, EMPs are pro-survival by carrying away pro-apoptotic mediators from ECs, while excessive EMPs under pathological conditions such as disturbed SS promote EC apoptosis by conveying pro-apoptotic mediators or signals back to ECs.

### EMPs on vasodilation

EMPs behave distinctly in modulating vasomotor tone under different conditions. For instance, EMP number was positively correlated with endothelium-dependent vasodilation in healthy controls, while significantly increased EMP levels were accompanied by markedly impaired vasodilation in the population with obesity (Dimassi et al., 2016). Analysis of EMP components provides some insights into this functional paradox. EMPs harbor functional eNOS as well as subunits of NAD(P)H oxidase. Hence, supplementation of EMPs is capable of restoring endothelium-dependent vasodilation in response to oxidative stress by promoting NO production, activating AKT signaling and suppressing ROS in ECs. These findings were consistent with another report that functional eNOS was detected in intact circulating microparticles (Mahmoud et al., 2017).

By contrast, sustained incubation of ECs with EMPs (at a dose of 10^6^/ml) alone diminished NO bioactivity and endothelial responsiveness (Mahmoud et al., 2017). EMPs carried a p22phox subunit of NAD(P)H oxidase, thereby blunting vasodilation through enhanced production of ROS (Brodsky et al., 2004). Similarly, microparticles from mice ischemic hind-limb muscle, of which 71% were CD144^+^ EMPs, expressed significantly higher levels of NADPH oxidase p47 (6-fold) and p67 subunits (16-fold) than controls (Leroyer et al., 2009).

Endothelium-dependent vasodilation is substantially impaired when SS is disturbed. Although few data are available regarding the role of EMPs on vasodilation under different patterns of SS, EMP production was found coexisting with vasodilation dysfunction. Disturbed SS markedly increased CD31^+^/CD41b^−^ EMPs level as well as reduced flow-mediated dilation (FMD) in chronic obstructive pulmonary disease (COPD) patients (Barak et al., 2017). In aldosterone-salt-induced hypertensive rat models, vascular remodeling and endothelial dysfunction were associated with increased circulating EMPs (Lopez Andres et al., 2012).

Therefore, the impact of EMPs on vascular tone is dependent on the comprised functional enzymes, the presence of oxidative stress and the concentration of EMPs. Whether EMPs play a compensatory or pathological role in endothelium-dependent vasodilation dysfunction under disturbed SS awaits further elucidation.

### EMPs on vascular inflammation

EMPs are capable of mediating vascular inflammation by conveying inflammatory mediators to nearby cells. EMPs derived from TNF-α-induced ECs were found to carry pro-inflammatory profiles including CCL-2, IL-6, IL-8, CXCL-10, CCL-5, TNF-α and ICAM-1. The inflamed EMPs mediated a selective transfer of functional inflammatory mediators to ECs and monocytes, thereby modulating their inflammatory status (Hosseinkhani et al., 2018). CD62e^+^ EMPs, which are under regulation by SS(Kim et al., 2015; Jung et al., 2017), were positively correlated with high-sensitivity C-reactive protein in pulmonary hypertension subjects (Amabile et al., 2009).

In regions of disturbed SS, inflammatory cytokines and cell adhesion molecules are increased, rendering these regions more prone to develop atherosclerosis. Lin et al. discovered EMPs were remarkably increased in patients with atrial septal defect and ventricular septal defect, which are associated with markedly enhanced disturbed SS. They further demonstrated that isolated EMPs promoted activation of P38 signaling pathway, and expression of TNF-α and IL-6 in cultured ECs (Lin et al., 2017). Therefore, EMPs may exert add-on effects to promote vascular inflammation in response to disturbed SS.

### EMPs on permeability

Existing evidence suggests that EMPs are involved in vascular barrier dysfunction. Densmore et al. showed that administration of exogenous EMPs collected from cultured ECs resulted in pronounced capillary leak and lung injury. The hyperpermeability effect of EMPs was further enhanced in a ‘second hit’ model by concomitant injection of lipopolysaccharide (Densmore et al., 2006).

Vascular permeability is increased in regions of disturbed SS, which enables macromolecules, such as low-density lipoproteins, to transport across endothelium monolayer and deposit in the subendothelial space (Conklin et al., 2007). In a chronic cerebral ischemia model by bilateral common carotid artery ligation that produces disturbed SS proximal to the ligation site (Winkel et al., 2015), isolated microparticles from the plasma caused a significant increase in endothelial barrier permeability mainly due to apoptosis (Edrissi et al., 2016). Hence, EMPs are likely to confer additional effects on impairing vascular permeability in response to disturbed SS.

### EMPs on coagulation system

EMPs carry von Willebrand factor (vWF) multimers, which promote platelet aggregation and increase the stability of the formed aggregates (Jy et al., 2005). Additionally, EMPs have been shown to promote EC thrombogenicity through tissue factor up-regulation, endothelial NO synthase downregulation and reduced NO-mediated inhibition of platelet aggregation (Abbas et al., 2017). Furthermore, Terrisse et al. found that EMPs from apoptotic human umbilical vein ECs were internalized into ECs, promoting ROS formation through xanthine oxidase and NADPH oxidase pathways, thus leading to increased vWF expression on EC surface and platelet aggregation (Terrisse et al., 2010).

When the shear rate of disturbed flow decreases to <100/s, a thixotropic increase in blood viscosity occurs in a non-Newtonian manner (Cho and Kensey, 1991). Under such circumstance, the low fluid shearing forces are not sufficient to overwhelm the forces associated with cell-cell interactions between platelets, erythrocytes, and leukocytes, thereby leading to increased vascular resistance and blood sludging (Hathcock, 2006). Meanwhile, disturbed SS at sites downstream of a stenotic site aggravates local platelet aggregation in a strongly vWF-dependent manner (Westein et al., 2013). Therefore, elevated EMPs under disturbed SS may further contribute to local hypercoagulation either by presenting vWF or enhancing platelet aggregation.

## Summary and perspective

**FIGURE 1:**
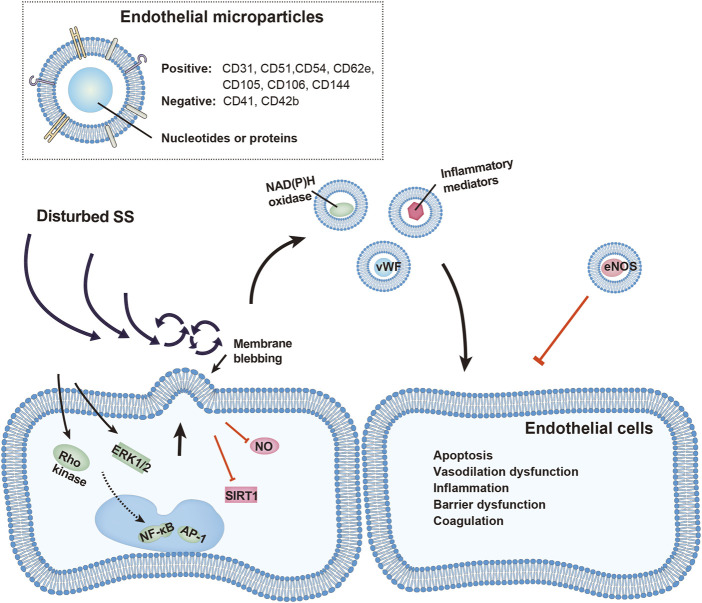
Schematic representation of vascular injury by EMPs under disturbed shear stress (SS). EMP release is increased under disturbed SS through activation of Rho kinase, ERK1/2, NF-κb, and AP-1 pathways, or by inhibition of STIR1 and nitric oxide (NO). Released EMPs, identified by presence of endothelial and absence of platelet surface markers, carry functional proteins including von Willebrand factors, NAD(P)H oxidase, inflammatory mediators and eNOS. These EMPs affect nearby endothelial cells and promote vascular apoptosis, vasodilation dysfunction, inflammatory response, barrier dysfunction and local hypercoagulation.

Fluid SS tightly regulates the release and function of EMPs, which are broadly involved in the modulation of cell survival, function, and cell-cell interactions. EMP release is remarkably increased under disturbed SS, which not only reflects EC activation but also modulates vascular senescence, dilation, inflammatory response, barrier function and local coagulation system via conveying functional enzymes and inflammatory mediators (Figure 1). These effects by EMPs profoundly shape the injurious impact of disturbed SS or other pro-inflammatory stimuli on the vasculature. By contrast, steady SS maintains vascular homeostasis by restricting EMP release. Future studies are warranted to explore the casual relationship between specific EMPs and the homeostasis and injury of the vasculature under different SS profiles. Targeting EMPs may serve as a potential strategy in alleviating disturbed SS-induced endothelial dysfunction and the development of atherosclerosis.
